# Editorial: Host plant resistance mechanisms against fungal pathogens

**DOI:** 10.3389/fpls.2022.1103046

**Published:** 2022-12-08

**Authors:** Rajtilak Majumdar, Kanniah Rajasekaran, Martha M. Vaughan, Peggy Ozias-Akins

**Affiliations:** ^1^ Northwest Irrigation and Soils Research, United States Department of Agriculture-Agricultural Research Service, Kimberly, ID, United States; ^2^ Food and Feed Safety Research Unit, Southern Regional Research Center, United States Department of Agriculture-Agricultural Research Service, New Orleans, LA, United States; ^3^ Mycotoxin Prevention and Applied Microbiology Research Unit, National Center for Agricultural Utilization Research, United States Department of Agriculture-Agricultural Research Service, Peoria, IL, United States; ^4^ Genetic and Genomics and Department of Horticulture, Institute of Plant Breeding, University of Georgia, Tifton, GA, United States

**Keywords:** plant disease resistance, susceptibility, fungi, genomics, mRNAs, proteomics, metabolomics, next generation sequencing (NGS)

Crop plants are constantly exposed to diverse biotic stressors during their lifetime. Fungal pathogens represent a predominant biotic stress of crops and account for 80-85% of known diseases leading to significant yield losses. Host plant resistance against fungal pathogens is due to diverse factors such as plant genetic background, physiological status, agroecological, and environmental conditions. In addition, the microbiome associated with host plants has also been shown to contribute to resistance by producing metabolites that modulate host plant defense pathways or exhibit antimicrobial properties. The advancement in next-generation sequencing (NGS) technology for genome/RNA sequencing and modern omic approaches such as proteomics, metabolomics, and interactomics have profusely helped to define host plant resistance mechanisms. These technological advances have made possible introgression of resistance traits into agronomically important varieties by traditional or molecular breeding. Recently, the application of highly sophisticated biotechnological tools using RNAi or CRISPR-Cas9-based gene editing has enabled us to precisely manipulate the integration and expression of key genes for enhanced host resistance. This special issue compiles articles that highlight mechanisms underlying host plant-fungal interactions that govern susceptibility or resistance to fungal pathogens including *Botrytis*, *Colletotrichum*, and *Fusarium*.

Gray mold disease in strawberry caused by the necrotrophic fungus *Botrytis cinerea* (*B. cinerea*) negatively impacts strawberry production worldwide. The fungus preferentially infects flowers and fruits of strawberry. Using *B. cinerea* resistant and susceptible cultivars and global mRNA sequencing, Xiao et al. demonstrated higher expression of defense-related genes and lower expression of genes associated with cell wall degrading enzymes and peroxidases in the resistant cultivar during early stages of infection. The authors also showed increased expression of calcium signaling pathway related genes namely *CPK*s, *RBOHD*s, *CNGC*s, and *CML*s and genes associated with jasmonic acid, auxin, and phenylpropanoid metabolism in the resistant cultivar. The work presented here could be useful for future breeding efforts or transgenic manipulation of candidate genes to improve gray mold resistance in strawberry.

In another study addressing strawberry leaf resistance against *B. cinerea*, Zhao et al. delineated the molecular basis of resistance. Using resistant and susceptible genotypes and a combination of tools including gene expression, quantification of metabolites, and microscopy, the authors dissected leaf-associated factors in woodland strawberry potentially contributing to resistance against the fungus. Higher basal (in absence of the fungus) expression of resistance related genes rather than induction of genes in response to the fungus was observed in the resistant genotype. Metabolites such as total phenolics, total flavonoids, glucose, galactose, citric acid, and ascorbic acid were positively correlated with *B. cinerea* resistance, whereas H_2_O_2_ and sucrose were negatively correlated with resistance. The authors suggested higher innate antioxidant profile of leaves as one of the key factors contributing to resistance against the fungus.

In plants thaumatin-like proteins (TLPs) have been implicated in a wide range of physiological processes including stress response and growth and development. The role of this gene family in Qingke (*Hordeum vulgare* L. var. *nudum*), Tibetan hulless barley grown in higher elevation regions, is not fully understood. Using genome and transcriptome mining through bioinformatics, Wang et al. demonstrated the role of specific members of the *TLP* gene family in biotic (powdery mildew) and abiotic (drought, low temperature) stress tolerance. Analysis of promoter regions of this gene family showed presence of putative transcription factor binding motifs associated with growth and development, hormone signaling, light and stress responses. Gene expression analysis using qRT-PCR validated the induction of specific members of *TLP*s in response to sodium salicylate and methyl jasmonate treatments. The work presented here provides future opportunities for crop improvement in Qingke which is a staple crop in Tibet.

Wheel wingnut (*Cyclocarya paliurus*; *C*. *paliurus*) is an economically important tree species and highly susceptible to the fungal pathogen *Colletotrichum fructicola*. Zheng et al. dissected the role of specific plant secondary metabolites in resistance against this fungus using *C*. *paliurus* resistant and susceptible cultivars. Through multiomic approaches namely mRNA sequencing and metabolomic analyses, the authors demonstrated that early induction and reprogramming of the flavonoid biosynthetic pathway potentially contribute to resistance to *C. fructicola*. The information presented here provides valuable information on biomarker/s for future resistance breeding in *C. paliurus* and/or using natural flavonoid extracts from a resistant cultivar as an antifungal treatment against this fungus.

Zheng et al. demonstrated the role of compatible allelopathic interactions in the rhizosphere reducing *Fusarium* wilt symptoms in faba bean (*Vicia faba* L.). The authors found that faba bean–wheat intercropping under field conditions significantly improved *Fusarium* wilt disease severity in comparison to monoculture of faba bean plants. Reduction in cellular contents of H_2_O_2_ and O_2_ accompanied by increased expression and activities of superoxide dismutase and catalase enzymes in the roots of faba bean plants were observed when intercropped with wheat. In addition, reduced benzoic acid and cinnamic acid in the rhizosphere soil of faba bean plants improved the stability of root cells. The authors used a reverse approach and showed that exogenous application of benzoic acid and cinnamic acid to the roots of faba bean plants increases *Fusarium* wilt symptoms under greenhouse conditions. This demonstrates the role of allelopathic compounds in the rhizosphere of faba bean crop in resistance to *Fusarium* wilt.

Overall, the work presented in this volume advances our understanding towards plant-fungal interactions and sets a good foundation for future breeding approaches to improve crop resistance to fungal pathogens.

## Author contributions

All authors listed have made substantial, direct, and intellectual contribution to the work and approved it for publication.

